# Minimally Invasive Extraction System Benex—Clinical Evaluation and Comparison

**DOI:** 10.3390/dj12080234

**Published:** 2024-07-24

**Authors:** Lyubomir Chenchev, Vasilena Ivanova, Krikor Giragosyan, Tasho Gavrailov, Ivan Chenchev

**Affiliations:** 1 Department of Oral Surgery, Faculty of Dental Medicine, Medical University of Plovdiv, 4000 Plovdiv, Bulgaria; vasilena.ivanova@mu-plovdiv.bg (V.I.); krikor.giragosyan@mu-plovdiv.bg (K.G.); 2 Center of Dental Implantology, Research Institute, Medical University of Plovdiv, 4000 Plovdiv, Bulgaria; ivan.chenchev@mu-plovdiv.bg (T.G.); tasho.gavrailov@mu-plovdiv.bg (I.C.)

**Keywords:** tooth extraction, atraumatic extraction, minimally invasive, dental implantology

## Abstract

Tooth extraction is one of the oldest and most well-known surgical procedures in dental medicine. It is still routinely performed by general practitioners and dental undergraduates. The Benex extraction system allows for the extraction of teeth in a vertical direction, which avoids most trauma against surrounding alveolar bone and soft tissues. The study included 56 patients who were recruited from the Department of Oral Surgery, Medical University—Plovdiv. The patients were split into two groups of 28 patients—Group I (control group) and Group II (study group). For each group, the success of the extraction, buccal cortical plate preservation, pain experience and early wound healing were assessed. There was no statistical significance between the success of the extractions in both groups. The Benex extractions preserved the buccal cortical plate in 95% of the cases, whereas the forceps extractions preserved it in only 71.8%, which is statistically significant. On the seventh day, patients in Group II reported less pain, without a significant difference. There was a significantly bigger number of completely healed extraction wounds on the 10th day. Atraumatic extractions allow for more hard and soft tissues to be preserved in the extraction site. This is essential for a successful outcome and the aesthetically pleasing results of the following dental restoration.

## 1. Introduction

Tooth extraction (*exodontia*) is one of the oldest and most well-known surgical procedures in dental medicine. It is still routinely performed to date by general practitioners, oral surgeons and dental undergraduates [1]. Dental implantology has also become a prime solution for the treatment of partial or complete tooth loss. Teeth, which would otherwise be saved by some dubious or hard-to-perform conservative treatment techniques, are now considered for extraction and implant replacement [2].

The classical means for tooth extraction—forceps and elevators—do not make it impossible to perform an atraumatic extraction. However, their use oftentimes leads to unexpected complications and unplanned difficulties during the procedure. Even for the more experienced practitioners, this may easily turn out to be a challenging extraction, requiring a not-so-atraumatic approach to fully complete. This leads to a varying extent to postoperative defects in the alveolar bone of the extraction site [3,4].

Post-extraction wound healing is a great indicator of how well the body recovers from the damage caused. This can occur with mild to no discomfort at all but can also be delayed and accompanied by different symptoms such as swelling, trismus, infection and a varying degree of pain, all of which can be signs of postoperative complications [5]. Decreasing the degree of trauma can not only prevent complications but is necessary to preserve bone and soft tissues [6].

Dental practitioners, and especially dental implantologists, are searching for a more predictable and less traumatic way to extract teeth. Different techniques, approaches and instruments are presented all the time, with varying degrees of utility, applicability and success. This is especially true in recent years, where the attention has been almost completely turned to immediate implant placement and loading.

The Benex extraction system is one such modern device that allows for the extraction of teeth in a vertical direction, to which teeth are least resistant. This avoids most, if not all, trauma against the surrounding alveolar bone and soft tissues [7]. This can potentially allow for more bone to be preserved, not only because of the lack of immediate damage but also due to the limited loss of osteoblast cells [8]. In this way, it is expected that sufficient tissues will be present for immediate treatment, and the following natural healing response will be unaffected and complete with lasting results.

## 2. Materials and Methods

### 2.1. Study Design

This study was a crossover randomized control trial.

The study was aimed at assessing the applicability of the modern Benex extraction system and comparing its efficacy in terms of several aspects against the conventional extraction forceps in the early wound healing period. The immediate trauma, such as cortical plate fracture, is determinant for the ultimate amount of bone loss and soft tissue recontouring, while pain and wound healing are indicators of the initial trauma, which is determinant for the success of immediate restorative procedures. All clinical procedures were performed by a single clinician to exclude possible bias and experience difference.

**Figure 1:**
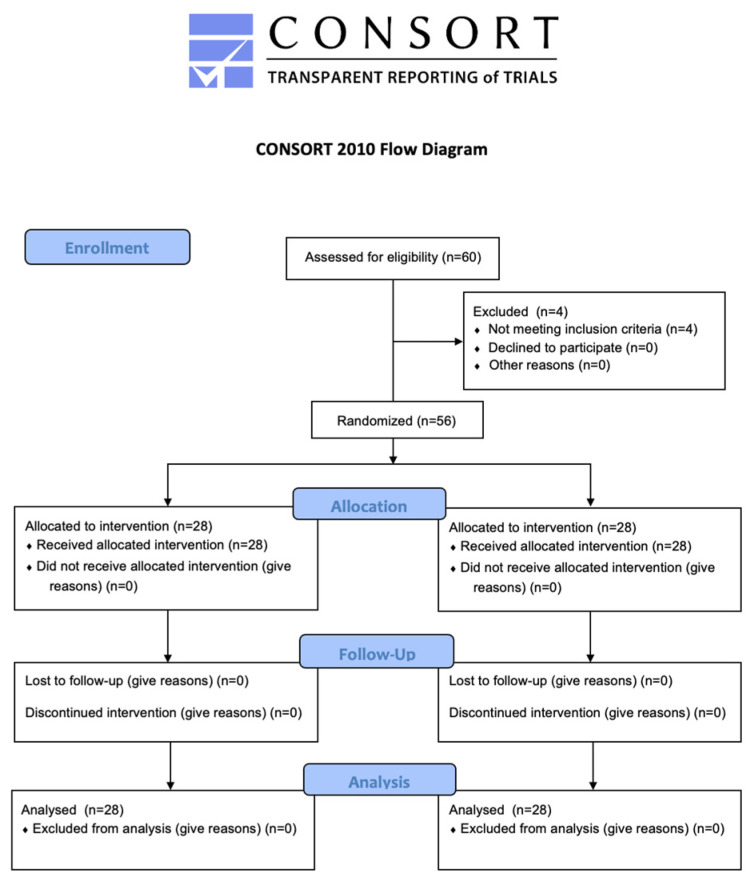
CONSORT 2010 flow diagram.

The study included 56 patients who were recruited from the Department of Oral Surgery at the Faculty of Dental Medicine, Medical University—Plovdiv and Center of Dental Implantology, Research Institute Medical University—Plovdiv. The study was approved by the ethics committee of the Medical University—Plovdiv. The study was registered in the NCT06496906 Registry of Clinical Trials of ClinicalTrials.gov and followed the guidelines described in the CONSORT 2010 (Consolidated Standards of Reporting Trials) statement on clinical trials—Figure 1.

The study was conducted over 12 months (January 2022–January 2023), and the procedures followed were in accordance with the Declaration of Helsinki, the ethical standards of the responsible committee on human experimentation and the rules of good practice in biomedical and educational research. All patients were informed about the nature and purpose of the study, and each of them signed an informed consent document granting permission for the dental procedures and the sampling of biological material.

The follow-up period of our study is limited by the fact that most minimally invasive techniques are used when immediate restorations are planned. For stable long-term results, clinicians still mostly consider other proven ridge preservation techniques.

### 2.2. Subjects and Sample

The patients were divided into two equal groups using block randomization, where every next patient was assigned to the group with the fewest subjects. A convenience sampling method was used to determine the number of participants. Group I included 28 patients whose teeth were extracted with conventional tooth extraction forceps and was considered the control group, while 28 patients were included in Group II, who had an extraction with the vertical extraction system Benex and were considered the study group. The study population consisted of males and females with a mean age of 37.2 ± 8.1 years, all above 18 years of age.

Any tooth with indication for closed extraction was considered. The condition of the teeth, whether treated endodontically or not, was not considered, because it should be of no difference for the final treatment plan, which includes a prosthetic or implant restoration.

Inclusion criteria:

- Patients with teeth indicated for closed extraction;
- Teeth with at least 1 mm of hard tooth tissue above the bone level;
- Patients without contraindications for surgical intervention (ASA 1 or 2);
- Patients with good oral hygiene.

Exclusion criteria:

- Patients with severe or uncontrolled systemic conditions or chronic immunosuppression;
- Teeth with mobility greater than Grade 1;
- Teeth indicated for open surgical extraction;
- Patients with acute odontogenic and oral infections;
- Drug or alcohol abuse;
- Patients on anticoagulant or antiaggregant drugs refusing to undergo prior tests and preparation for tooth extraction;
- Patients on radiotherapy, chemotherapy or oral bisphosphonates for up to 6 months before the procedure;
- Patients with psychiatric conditions.

### 2.3. Clinical Procedure

Anaesthesia was performed under infiltration with 4% articaine hydrochloride with adrenaline (dilution, 1:100,000; Septodont, Saint-Maur-des-Fossés, France). An impression with Zetaplus putty C-silicon Zetaplus (Zermack, Badia Polesine (Rovigo), Italy) was made with the impression tray included in the Benex extraction kit (Benex extractor, Hager & Meisinger GmbH, Neuss, Germany). Periodontal ligament fibers were cut using a scalpel blade #15. 

The Benex system is quite different from the instruments most people are used to and consists of the Benex extractor itself, diamond burs, pull ropes, self-tapping retention screws and a sectional impression tray [9].

Drilling was performed on the long axis of the tooth with the diamond-coated twist drill from the kit. The pull rope retention screw was inserted, and the impression tray was repositioned as a support disk. Then, the pull rope was attached to the retention screw, passed over the roller and fixed in an appropriate notch of the extractor.

At this point, attention should be paid to the fact that the pulling of the tooth should occur in as close to a vertical direction as possible. Any misplacement of the components of the extraction system can easily lead to failure.

**Figure 2:**
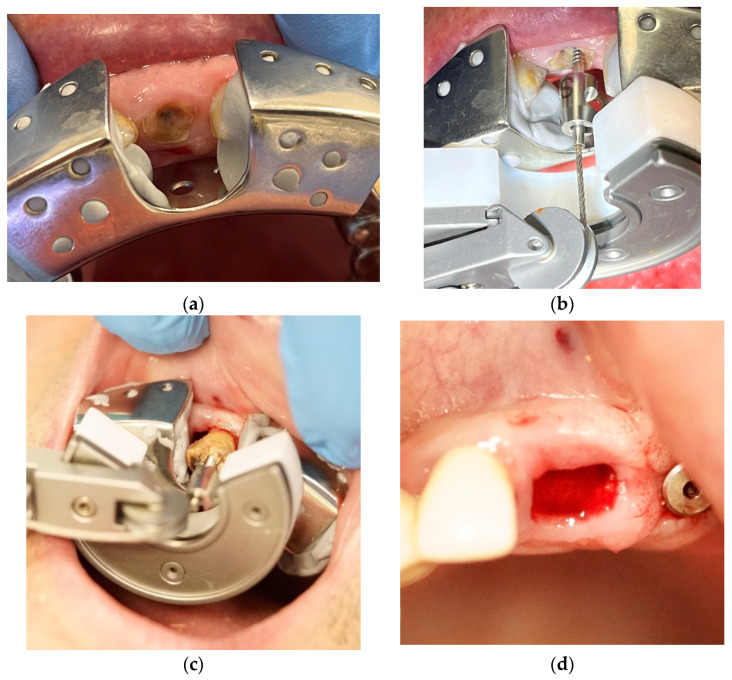
Benex tooth extraction: (**a**) Impression and positioning of the impression tray; (**b**) Assembled Benex extractor; (**c**) Tooth extraction process; (**d**) Final socket extraction wound.

Extraction was then accomplished by gradually turning the hand screw in a clockwise direction. Debridement of the wound was then carried out, as needed. The extraction sockets were not filled with any resorbable material and were left to normal physiological healing by secondary intention. No sutures were placed over the wounds either (Figure 2).

When extracting multi-rooted teeth, the roots of the tooth were separated before the extraction and extracted separately. To achieve the same level of difficulty between the two techniques, the roots were also separated for the extractions with conventional extraction forceps, although this is normally not a mandatory requirement for the technique.

### 2.4. Evaluation Method

#### 2.4.1. Success of the Extraction Procedure

Extraction success was graded from 1 to 5 based on the scale of Choi et al. [10] and its later modification by Patel et al. [11]:

- Complete success (Score 5): extraction without crown and root fracture;
- Limited success with root tip fracture (Score 4): extraction involving root tip fracture;
- Limited success with root fracture (Score 3): extraction involving one or more root fracture or crown fracture;
- Limited success with osteotomy (Score 2): fracture-free extraction and partial osteotomy in case divergent roots and a thick cortical bone were present;
- Failure (Score 1): failure to extract.

A failed extraction was every extraction that was not finished off with the instruments for the given group and/or resulted in an open flap extraction and was rated with Score 1. Any other extraction was rated based on the scale presented above and included in the group count.

The results between the different groups were compared using an independent-samples *t*-test. The results are presented as the mean-and-individual-values.

#### 2.4.2. Buccal Cortical Plate Preservation

**Figure 3:**
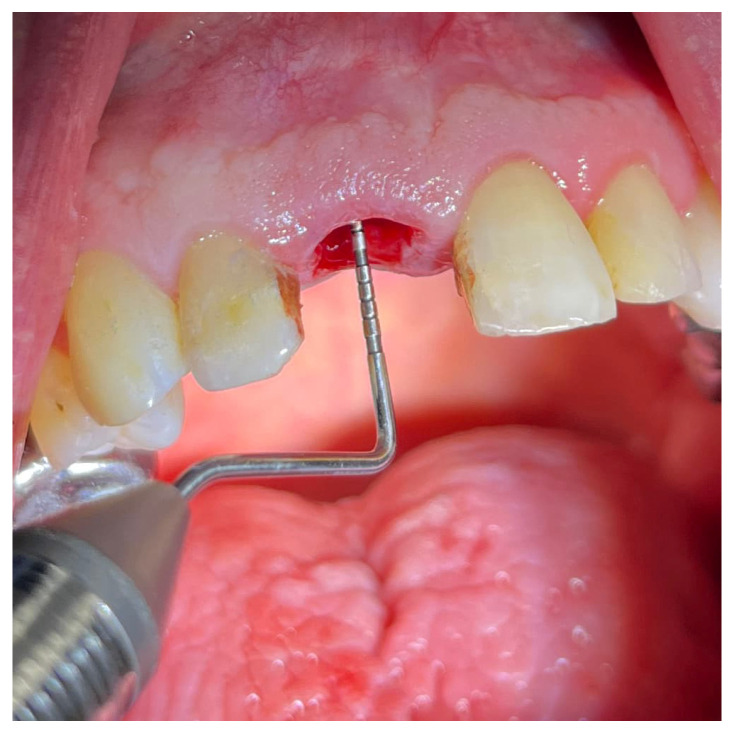
Evaluation of the buccal cortical plate level after a tooth extraction.

The amount of buccal cortical plate that was preserved or lost during the extraction was scored as preserved (no difference between the initial and post-extraction height), partially preserved (up to 4 mm loss after the extraction) and missing (over 4 mm loss after the extraction). The amount of lost buccal cortical plate was calculated by the difference between the preoperative and postoperative probing depth on the buccal side of the socket (Figure 3). This is similar to the recently presented single-rooted tooth extraction wound classification by Hamoun et al. [12], where they measure the missing buccal cortical plate in percentages. Our results were aggregated in cross-tabulations and were analyzed with a Chi-square test.

#### 2.4.3. Subjective Pain Experience

Pain was rated on a VAS scale on a piece of paper and measured in centimeters on the day of extraction (day 0) and the 1st, 3rd and 7th days following the extraction procedure. The patients were asked to place a cross on the linear scale where pain intensity experience on the given day increases from left to right. The results were analyzed with a single factor repeated measures ANOVA model.

#### 2.4.4. Early Wound Healing

Wound healing was scored on the 3rd, 7th and 10th days after the extraction. It was based on Landry’s index (LWHI—Landry Wound Healing Index), also known as the Landry, Turnbull and Howley index [13]. The index performs a complex evaluation of the extraction socket, taking into consideration the wound size, the surrounding tissue color, bleeding on palpation, the presence of granulation tissue, the presence of pus and the gingival margin status. The results were compared using the nonparametric Mann–Whitney U test.

## 3. Results

**Figure 4:**
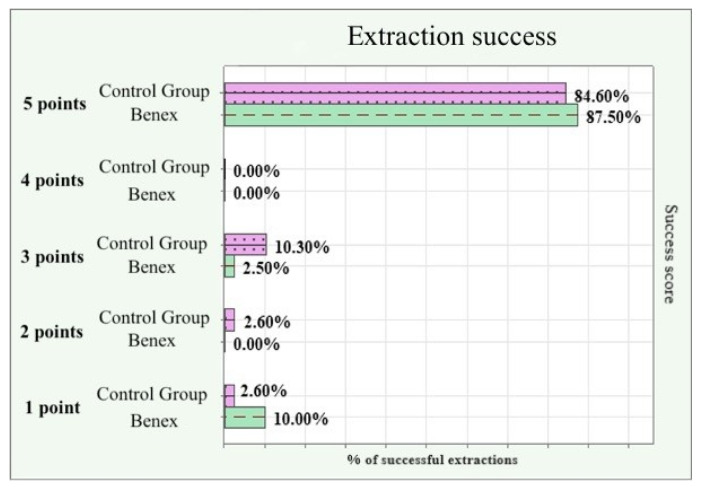
Comparison of extraction success rate % for both groups.

Figure 4 presents the success of the extractions in the control group and the study group graded on a scale from 1 (unsuccessful) to 5 (full success). The number of completely successful extractions (5 points) is almost equal in both groups. There were slightly more failures (1 point) in the study group, but there were equally as many partial failures (2–4 points) in the control group. The average success of the extractions with the Benex extractor was 4.61 points, while the average for the classical instruments was 4.55 points. There was no statistical significance between the two groups in terms of the number of successful and failed extractions.

**Figure 5:**
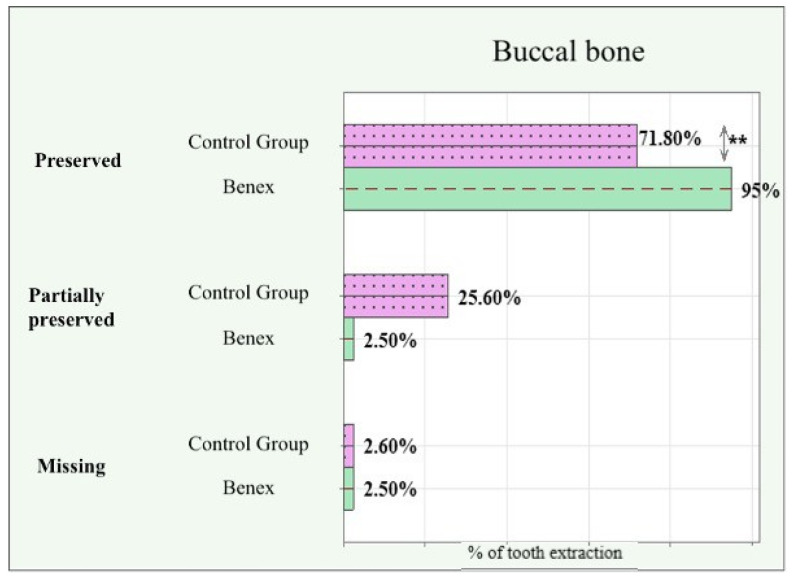
Buccal cortical plate preservation—preserved (no loss); partially preserved (loss < 4 mm); missing (loss > 4 mm). **: significant difference.

The preservation of buccal cortical bone can be seen in Figure 5. There was a statistically significant difference in the number of cases where the buccal cortical plate remained intact and completely preserved in the study group. The Benex extractions preserved the buccal cortical plate in 95% of the cases, whereas the forceps extractions preserved it in only 71.8%. About one-fourth of all extractions in the control group had a partial fracture (<4 mm) of the buccal cortical plate. Both groups had an equal number of extractions completely miss the buccal cortical plate (>4 mm).

The results for the subjective pain intensity score measured in centimeters on VAS are presented in Table 1. Both groups show a spike in the pain in the first day after the extraction and gradual improvement in the following days. While the study group shows marginally better results, there is no statistically significant difference.

The healing score based on Landry’s wound healing index (LWHI) can be seen in Table 2. The Benex extractions showed better healing in all periods, where 100% of the extraction wounds had healed in a Very Good or Excellent manner. Only 78.6% of the extractions in the control group had Very Good or Excellent healing scores.

## 4. Discussion

Dental specialists are looking for ways to predictably improve the success of their extractions while reducing the amount of trauma. The latter is directly correlated with how much hard and soft tissues will be affected and lost during the healing period [14]. A variety of socket and ridge preservation techniques exist, aimed at reducing the overall bone loss after a tooth extraction. They, however, do not overrule the benefits of atraumatic extraction [15,16].

Muska et al. [17] performed 111 extractions with the Benex extractor, with an overall success rate of 83%. Furthermore, based on their judgement, about 44% of all teeth would have otherwise required a surgical extraction, which is often not a simple procedure. Hong et al. [7] extracted a total of 323 teeth with Benex, where 46 were included after a previously unsuccessful extraction with extraction forceps and have a success rate of 85.4%. They extended their study further and performed 1719 extractions with extraction forceps and found 1 out of 5 (22%) cases to require surgical extraction, whereas with the use of Benex, only 1 of 20 (6%) required a surgical extraction. In our study, we did not limit the inclusion criteria to teeth with tissues above the bone level but did not include teeth that were directly indicated for open flap surgery before the start of the procedure.

The average success of the Benex extractions in our study was calculated to be 4.61 points, while the same for the classical instruments was 4.55. This shows a marginally better outcome for the Benex extractions. Hong’s findings may partially explain the greater extraction success of the Benex device in comparison to the classic extraction forceps. It seems that the Benex extractor excels when it comes to difficult-to-extract teeth with roots below the level of the surrounding bone or cases of previously failed extraction with the conventional means.

In our study, we included both single- and multi-rooted teeth. While there was success with maxillary first premolars, the extraction of molars was incredibly difficult due to the bulk of the Benex device, even after separating their roots. This coincides with the findings of Muska et al. [17], who reported a success rate of 89% for teeth with a single root, in comparison to one of only 43% in multi-rooted teeth. Hong et al. [7] also conclude that the odds of failure in multi-rooted teeth are about 2.2 times greater.

In our study, we found that teeth with previous endodontic treatment were more difficult to extract with Benex and had a higher chance of failure. This is possibly because the root canals were already weakened during the endodontic filing and shaping. Furthermore, such teeth were oftentimes left exposed for a long period of time, and decay or caries could be found inside the root canals. This does not allow the Benex extraction post to be firmly screwed inside the prepared root. This is in concordance with the results of Hong et al. [7], who reported that the failure rate in endodontically treated teeth was 2.1 times higher.

Another possibility for failure is the improper fixation of the root canal screw-post. Muska et al. [17] found that the second major type of failure was due to the insufficient retention of the screw due to operator misjudgment or previously existing fractures and/or caries. However, we noticed that the odds of success can be somewhat improved if the screw penetrates the entire tooth and reaches or slightly engages the bone on the opposing end. This is also supported by Krug et al. [18], who found perforation in 3 of 51 cases, with no lasting damage and complete healing of the periodontium after the extrusion.

Most dental extractions normally heal with mild to no symptoms. However, sometimes the postoperative period can be accompanied by discomfort and different symptoms, including varying degrees of pain, temperature and fever, and even lead to life-threatening situations. Compromised wound healing will not only result in patient complaints and additional appointments but can complicate and even make a following tooth replacement impossible [19].

In our study, we followed up the extractions for up to 10 days and evaluated the extraction wound healing using the LWHI. There was a statistically significant difference in favor of the Benex extractions. Makki et al. [20] used the same index to follow up the patients up to 4 weeks after, and their results at the end of this period show that 94.7% of all Benex extraction wounds had healed with a score of 5 or 4, while the extraction forceps had 47.4%. The difference between the success of the extractions with Benex and the extraction forceps matches the findings of our study.

It is a well-established fact that after a tooth extraction, the hard and soft tissues undergo remodeling and are ultimately reduced [21]. The loss of a tooth leads to the initiation of resorption processes, which mainly affect the bone on the buccal side of the extraction site. This bone loss is correlated with the amount of trauma caused during the extraction and the destruction of the existing stem cells in the periodontal space [22]. Even with the use of different techniques for ridge preservation, the soft tissues, too, do not remain unaffected with a predominant change in their buccal contour [23]. The following bone loss makes implant placement a challenging endeavor, while a subsequent soft tissue recession may expose an already placed implant and compromise its aesthetics and life expectancy.

The fact that the Benex system extracts a tooth in a vertical direction puts out an expectation that it is more sparing to the buccal cortical plate. In our study, the buccal cortical plate remained intact in 95% of the Benex extractions, compared with only 71.8% for the extraction forceps. We did not find another study in the available literature to compare our results with. Furthermore, the Benex system requires no luxation motions, which causes no additional compression and expansion of hard tissues or deformation and tearing of soft tissues. This is backed by the findings that the actual force and bone density are not as important for the success of a tooth extraction as how this force is applied and the technique used [23].

## 5. Conclusions

Atraumatic extractions allow for more hard and soft tissues to be preserved in the extraction site. The Benex extractor is a device that pulls the tooth or root out of the socket, eliminating any trauma to the surrounding bone and causing minimal to no negative effect on the surrounding soft tissues and their contour. This is all crucial for the successful outcome and aesthetically pleasing results of a following fixed partial denture or implant restoration. The Benex device, after some initial getting used to, is a tool used to predictably achieve these results, even in cases otherwise considered for open surgical extractions. Its benefits might include:

- Elimination of trauma to surrounding bone and soft tissue;
- Practically non-existent buccal cortical plate fractures;
- Improved early wound healing;
- More predictable results in immediate restorations.

However, more extensive research with a longer follow-up period is needed to verify this.

Limitations: A limitation of our study is the short follow-up period, which only evaluates the immediate effect of the trauma and not the long-term effect of the extraction on the hard and soft tissues and their volume. Furthermore, the sample size is limited and might have an impact on the ultimate credibility of the presented results.

## Figures and Tables

**Table 1:** VAS-measured pain intensity score for 7 days after the extraction.

Pain Score	Benex Mean (SD)	Conventional Instruments Mean (SD)	*p* Cross-Group Comparison
Day 0	0.81 (0.73)	0.83 (0.46)	0.897
Day 1	1.02 (0.43)	1.26 (0.61)	0.103
Day 3	0.60 (0.74)	0.95 (1.37)	0.241
Day 7	0.06 (0.15)	0.26 (0.37)	0.016

**Table 2:** Early wound healing score. Clinical patient follow-up for a 10-day period.

Early Wound Healing Score	Benex n = 28	Conventional Instruments n = 28	*p* Cross-Group Comparison
**Day 3**			
Very Poor	0.00% (0)	0.00% (0)	0.555
Poor	**85.70%** (24)	**78.60%** (22)	
Good	7.10% (2)	17.90% (5)	
Very Good	7.10% (2)	3.60% (1)	
Excellent	0.00% (0)	0.00% (0)	
**Day 7**			
Very Poor	0.00% (0)	0.00% (0)	0.694
Poor	25.00% (7)	28.60% (8)	
Good	28.60% (8)	35.70% (10)	
Very Good	39.30% (11)	21.40% (6)	
Excellent	7.10% (2)	14.30% (4)	
**Day 10**			
Very Poor	0.00% (0)	0.00% (0)	0.041
Poor	0.00% (0)	14.30% (4)	
Good	0.00% (0)	7.10% (2)	
Very Good	28.60% (8)	28.60% (8)	
Excellent	71.40% (20)	50.00% (14)	

## Data Availability

Study was performed as part of a university project. Data can be requested directly from one of the authors, but will not be publicly available, however.
